# Update: Public Health Response to the Coronavirus Disease 2019 Outbreak — United States, February 24, 2020

**DOI:** 10.15585/mmwr.mm6908e1

**Published:** 2020-02-28

**Authors:** Daniel B. Jernigan, CDC COVID-19 Response Team

**Affiliations:** 1 CDC COVID-19 Response Team, CDC.

An outbreak of coronavirus disease 2019 (COVID-19) caused by the 2019 novel coronavirus (SARS-CoV-2) began in Wuhan, Hubei Province, China in December 2019, and has spread throughout China and to 31 other countries and territories, including the United States (*1*). As of February 23, 2020, there were 76,936 reported cases in mainland China and 1,875 cases in locations outside mainland China (*1*). There have been 2,462 associated deaths worldwide; no deaths have been reported in the United States. Fourteen cases have been diagnosed in the United States, and an additional 39 cases have occurred among repatriated persons from high-risk settings, for a current total of 53 cases within the United States. This report summarizes the aggressive measures (*2*,*3*) that CDC, state and local health departments, multiple other federal agencies, and other partners are implementing to slow and try to contain transmission of COVID-19 in the United States. These measures require the identification of cases and contacts of persons with COVID-19 in the United States and the recommended assessment, monitoring, and care of travelers arriving from areas with substantial COVID-19 transmission. Although these measures might not prevent widespread transmission of the virus in the United States, they are being implemented to 1) slow the spread of illness; 2) provide time to better prepare state and local health departments, health care systems, businesses, educational organizations, and the general public in the event that widespread transmission occurs; and 3) better characterize COVID-19 to guide public health recommendations and the development and deployment of medical countermeasures, including diagnostics, therapeutics, and vaccines. U.S. public health authorities are monitoring the situation closely, and CDC is coordinating efforts with the World Health Organization (WHO) and other global partners. Interim guidance is available at https://www.cdc.gov/coronavirus/index.html. As more is learned about this novel virus and this outbreak, CDC will rapidly incorporate new knowledge into guidance for action by CDC, state and local health departments, health care providers, and communities.

Person-to-person spread of COVID-19 appears to occur mainly by respiratory transmission. How easily the virus is transmitted between persons is currently unclear. Signs and symptoms of COVID-19 include fever, cough, and shortness of breath (*4*). Based on the incubation period of illness for Middle East respiratory syndrome (MERS) and severe acute respiratory syndrome (SARS) coronaviruses, as well as observational data from reports of travel-related COVID-19, CDC estimates that symptoms of COVID-19 occur within 2–14 days after exposure. Preliminary data suggest that older adults and persons with underlying health conditions or compromised immune systems might be at greater risk for severe illness from this virus (*5*).

## COVID-19 Cases in the United States

As of February 23, 14 COVID-19 cases had been diagnosed in the following six states: Arizona (one case), California (eight), Illinois (two), Massachusetts (one), Washington (one), and Wisconsin (one). Twelve of these 14 cases were related to travel to China, and two cases occurred through person-to-person transmission to close household contacts of a person with confirmed COVID-19. An additional 39 cases were reported among repatriated U.S. citizens, residents, and their families returning from Hubei province, China (three), and from the Diamond Princess cruise ship that was docked in Yokohama, Japan (36). Thus, there have been 53 cases within the United States. No deaths have been reported in the United States.

## CDC Public Health Response

As of February 24, 2020, a total of 1,336 CDC staff members have been involved in the COVID-19 response, including clinicians (i.e., physicians, nurses, and pharmacists), epidemiologists, veterinarians, laboratorians, communicators, data scientists and modelers, and coordination staff members. Of these CDC staff members, 497 (37%) have been deployed to 39 locations in the United States and internationally, including CDC quarantine stations at U.S. ports of entry, state and local health departments, hospitals, and U.S. military bases that are housing quarantined persons, as well as WHO and ministries of health around the world. CDC staff members are working with state, local, tribal, and territorial health departments and other public health authorities to assist with case identification, contact tracing, evaluation of persons under investigation (PUI) for COVID-19,* and medical management of cases; and with academic partners to understand the virulence, risk for transmission, and other characteristics of this novel virus.

CDC teams are working with the Department of Homeland Security at 11 airports where all flights from China are being directed to screen travelers returning to the United States, and to refer them to U.S. health departments for oversight of self-monitoring. CDC is also working with other agencies of the U.S. government including the U.S. Department of Defense; multiple operational divisions with the U.S. Department of Health and Human Services, including the Assistant Secretary for Preparedness and Response and the Administration for Children and Families; and the U.S. Department of State to safely evacuate U.S. citizens, residents, and their families to the United States from international locations where there is substantial, sustained transmission of COVID-19, and to house them and monitor their health during a 14-day quarantine period.

Specific guidance has been developed and posted online for health care settings, including for patient management; infection control and prevention; laboratory testing; environmental cleaning; worker safety; and international travel.^†^ Guidance is updated as more is learned. To prepare for the possibility of community spread of COVID-19, CDC has developed tailored guidance and communications materials for communities, health care settings, public health, laboratories, schools, and businesses. Chinese and Spanish versions of certain documents are available.

**Information for travelers.** Several recent travel notices have been posted by CDC to inform travelers and clinicians about current health issues that could affect travelers’ health.^§^ A Level 3 travel notice (avoid all nonessential travel) for China has been in effect since January 27. On February 19, Level 1 travel notices (practice usual precautions) for travelers to Hong Kong and Japan were posted. On February 22, the Level 1 travel notice for Japan was raised to Level 2 (practice enhanced precautions). A Level 2 travel notice was posted for South Korea on February 22, which was updated to Level 3 on February 24. Level 1 travel notices were posted for Iran and Italy on February 23, and then updated to Level 2 on February 24. In addition, CDC has posted information for travelers regarding apparent community transmission in Singapore, Taiwan, Thailand, and Vietnam, and recommendations for persons to reconsider cruise ship voyages in Asia.

**Airport screening.** As of February 23, a total of 46,016 air travelers had been screened at the 11 U.S. airports to which all flights from China are being directed. Since February 2, travelers to the United States who have been in China in the preceding 14 days have been limited to U.S. citizens and lawful permanent residents and others as outlined in a presidential proclamation.^¶^ Incoming passengers are screened for fever, cough, and shortness of breath. Any travelers with signs or symptoms of illness receive a more comprehensive public health assessment. As of February 23, 11 travelers were referred to a hospital and tested for infection; one tested positive and was isolated and managed medically. Seventeen travelers were quarantined for 14 days because of travel from Hubei Province, China, an area that was designated as high risk for exposure to COVID-19**; 13 of these 17 have completed their quarantine period.

**Persons under investigation (PUIs).** Recognizing persons at risk for COVID-19 is a critical component of identifying cases and preventing further transmission. CDC has responded to clinical inquiries from public health officials, health care providers, and repatriation teams to evaluate and test PUIs in the United States for COVID-19 following CDC guidance. As of February 23, 479 persons from 43 states and territories had been or are being tested for COVID-19; 14 (3%) had a positive test, 412 (86%) had a negative test, and 53 (11%) test results are pending.

**Laboratory testing.** As part of laboratory surge capacity for the response, CDC laboratories are testing for SARS-CoV-2 to assist with diagnosis of COVID-19. During January 18–February 23, CDC laboratories used real-time reverse transcription–polymerase chain reaction (RT-PCR) to test 2,620 specimens from 1,007 persons for SARS-CoV-2. Some additional testing is performed at selected state and other public health laboratories, with confirmatory testing at CDC. CDC is developing a serologic test to assist with surveillance for SARS-CoV-2 circulation in the U.S. population. The test detects antibodies (immunoglobulin [Ig]G, IgA, and IgM) indicating SARS-COV-2 virus exposure or past infection. In addition, CDC laboratories are developing assays to detect SARS-CoV-2 viral RNA and antigens in tissue specimens. Finally, following CDC’s establishment of SARS-CoV-2 in cell culture, CDC shared virus isolates with the Biodefense and Emerging Infections Research Resources Repository to securely distribute isolates to U.S. public health and academic institutions for additional research, including vaccine development.

**Repatriation flights from areas with substantial COVID-19 transmission.** During January 29–February 6, the U.S. government repatriated 808 U.S. citizens, residents, and their families from Hubei Province, China, on five chartered flights. At the time of departure, all travelers were free of symptoms for COVID-19 (fever or feverishness, cough, difficulty breathing). After arriving in the United States, the repatriated travelers were quarantined for 14 days at one of five U.S. military bases. CDC and U.S. government staff members monitored these travelers’ health. As of February 23, 28 (3%) of these persons developed COVID-19-related symptoms and were evaluated for infection; three were found to be positive for SARS-CoV-2 and were referred for medical care and isolation. As of February 24, the remaining 805 travelers had completed their 14-day quarantine.

On February 3, passengers and crew of the Diamond Princess cruise ship were quarantined off Yokohama, Japan; a passenger who had recently disembarked in Hong Kong was confirmed to have COVID-19, and ongoing transmission was identified on the ship. By February 16, a total of 355 cases of COVID-19 had been identified among passengers and crew,^††^ including 67 U.S. citizens or residents. As a result, during February 16–17, the U.S. government assisted in the repatriation of 329 U.S. citizens or residents from the ship. These travelers returned on two chartered flights. As of February 23, 36 (11%) of these repatriated persons had tested positive for SARS-CoV-2 and are under appropriate medical supervision. The remaining repatriated persons are in quarantine for 14 days. CDC is working with the U.S. embassy in Japan and the Japanese government to support U.S. passengers and crew who remained in Japan.

## Discussion

COVID-19 is a serious public health threat. Cases of COVID-19 have been diagnosed in the United States, primarily in travelers from China and quarantined repatriates, and also in two close contacts of COVID-19 patients. Currently, COVID-19 is not recognized to be spreading in U.S. communities. If sustained transmission in U.S. communities is identified, the U.S. response strategy will enhance implementation of actions to slow spread in communities (*2*,*6*). Implementation of basic precautions of infection control and prevention, including staying home when ill and practicing respiratory and hand hygiene will become increasingly important.

Community-level nonpharmaceutical intervention might include school dismissals and social distancing in other settings (e.g., postponement or cancellation of mass gatherings and telework and remote-meeting options in workplaces). These measures can be disruptive and might have societal and economic impact on individual persons and communities (*6*). However, studies have shown that early layered implementation of these interventions can reduce the community spread and impact of infectious pathogens such as pandemic influenza, even when specific pharmaceutical treatments and vaccines are not available (*7*,*8*). These measures might be critical to avert widespread COVID-19 transmission in U.S. communities (*2*,*6*). Mitigation measures implemented in China have included the closing of major transport hubs and preventing exit from certain cities with widespread transmission, cancellation of Chinese New Year celebrations, and prohibition of attendance at school and work (*5*). However, the impact of these measures in China has not yet been evaluated.

In the United States, the National Institutes of Health (NIH) and their collaborators are working on development of candidate vaccines and therapeutics for COVID-19. In China, multiple clinical trials of investigational therapeutics have been implemented, including two clinical trials of remdesivir, an investigational antiviral drug.^§§^ An NIH randomized controlled clinical trial of investigational therapeutics for hospitalized COVID-19 patients in the United States was approved by the Food and Drug Administration; the first investigational therapeutic to be studied is remdesivir.^¶¶^ In the absence of a vaccine or therapeutic, community mitigation measures are the primary method to respond to widespread transmission and supportive care is the current medical treatment.

COVID-19 symptoms are similar to those of influenza (e.g., fever, cough, and shortness of breath), and the current outbreak is occurring during a time of year when respiratory illnesses from influenza and other viruses, including other coronaviruses that cause the “common cold,” are highly prevalent. To prevent influenza and possible unnecessary evaluation for COVID-19, all persons aged ≥6 months should receive an annual influenza vaccine; vaccination is still available and effective in helping to prevent influenza (*9*). To decrease risk for respiratory disease, persons can practice recommended preventive measures.*** Persons ill with symptoms of COVID-19 who have had contact with a person with COVID-19 or recent travel to countries with apparent community spread^†††^ should communicate with their health care provider. Before seeking medical care, they should consult with their provider to make arrangements to prevent possible transmission in the health care setting. In a medical emergency, they should inform emergency medical personnel about possible COVID-19 exposure. 

Areas for additional COVID-19 investigation include 1) further clarifying the incubation period and duration of virus shedding, which have implications for duration of quarantine and other mitigation measures; 2) studying the relative importance of various modes of transmission, including the role of droplets, aerosols, and fomites; understanding these transmission modes has major implications for infection control and prevention, including the use of personal protective equipment; 3) determining the severity and case-fatality rate of COVD-19 among cases in the U.S. health care system, as well as more fully describing the spectrum of illness and risk factors for infection and severe disease; 4) determining the role of asymptomatic infection in ongoing transmission; and 5) assessing the immunologic response to infection to aid in the development of vaccines and therapeutics. Public health authorities are monitoring the situation closely. As more is learned about this novel virus and this outbreak, CDC will rapidly incorporate new knowledge into guidance for action.

**Summary**

**What is already known about this topic?**

An outbreak of coronavirus disease 2019 (COVID-19) has spread throughout China and to 31 other countries and territories, including the United States.

**What is added by this report?**

Fourteen cases have been diagnosed in the United States, in addition to 39 cases among repatriated persons from high-risk settings, for a current total of 53 cases within the United States. The U.S. government and public health partners are implementing aggressive measures to slow and contain transmission of COVID-19 in the United States.

**What are the implications for public health practice?**

Interim guidance is available at https://www.cdc.gov/coronavirus/index.html. As more is learned about this virus and the outbreak, CDC will rapidly incorporate new knowledge into guidance for action.

## Footnotes

* Criteria to guide evaluation and testing of patients under investigation for SARS-CoV-2 include 1) fever or signs or symptoms of lower respiratory tract illness (e.g., cough or shortness of breath) in any person, including a health care worker, who has had close contact with a patient with laboratory-confirmed SARS-CoV-2 infection within 14 days of symptom onset; 2) fever and signs or symptoms of lower respiratory tract illness (e.g., cough or shortness of breath) in any person with a history of travel from Hubei Province, China, within 14 days of symptom onset; or 3) fever and signs or symptoms of lower respiratory tract illness (e.g., cough or shortness of breath) requiring hospitalization in any person with a history of travel from mainland China within 14 days of symptom onset. Additional information is available at https://emergency.cdc.gov/han/han00427.asp and https://emergency.cdc.gov/han/han00426.asp.

† https://www.cdc.gov/coronavirus/2019-ncov/index.html.

§ https://www.cdc.gov/coronavirus/2019-ncov/travelers/index.html.

¶ Office of the President. Proclamation on suspension of entry as immigrants and nonimmigrants of persons who pose a risk of transmitting 2019 novel coronavirus. Washington, DC: Office of the President; 2020. https://www.whitehouse.gov/presidential-actions/proclamation-suspension-entry-immigrants-nonimmigrants-persons-pose-risk-transmitting-2019-novel-coronavirus/.

** https://www.cdc.gov/coronavirus/2019-ncov/travelers/from-china.html.

†† https://www.who.int/docs/default-source/coronaviruse/situation-reports/20200216-sitrep-27-covid-19.pdf?sfvrsn = 78c0eb78_2.

§§ https://clinicaltrials.gov/ct2/show/NCT04257656?cond = remdesivir&draw = 2&rank = 1; https://clinicaltrials.gov/ct2/show/NCT04252664?cond = remdesivir&draw = 2&rank = 2.

¶¶
https://clinicaltrials.gov/ct2/show/NCT04280705?cond = COVID-19&draw = 4&rank = 22 .

*** https://www.cdc.gov/coronavirus/2019-ncov/about/prevention-treatment.html.

††† https://www.cdc.gov/coronavirus/2019-ncov/locations-confirmed-cases.html.
